# Effects of Heat Stress on Plant-Nutrient Relations: An Update on Nutrient Uptake, Transport, and Assimilation

**DOI:** 10.3390/ijms242115670

**Published:** 2023-10-27

**Authors:** Sasmita Mishra, Kim Spaccarotella, Jaclyn Gido, Ishita Samanta, Gopal Chowdhary

**Affiliations:** 1 Department of Biology, Kean University, 1000 Morris Avenue, Union, NJ 07083, USA; 2 Plant Molecular Biology Laboratory, School of Biotechnology, KIIT—Kalinga Institute of Industrial Technology, Bhubaneswar 751024, Odisha, Indiagkchowdhary@kiitbiotech.ac.in (G.C.)

**Keywords:** heat stress, nutrient uptake, nutrient-uptake proteins, peroxisomes, phytochemicals

## Abstract

As a consequence of global climate change, the frequency, severity, and duration of heat stress are increasing, impacting plant growth, development, and reproduction. While several studies have focused on the physiological and molecular aspects of heat stress, there is growing concern that crop quality, particularly nutritional content and phytochemicals important for human health, is also negatively impacted. This comprehensive review aims to provide profound insights into the multifaceted effects of heat stress on plant-nutrient relationships, with a particular emphasis on tissue nutrient concentration, the pivotal nutrient-uptake proteins unique to both macro- and micronutrients, and the effects on dietary phytochemicals. Finally, we propose a new approach to investigate the response of plants to heat stress by exploring the possible role of plant peroxisomes in the context of heat stress and nutrient mobilization. Understanding these complex mechanisms is crucial for developing strategies to improve plant nutrition and resilience during heat stress.

## 1. Introduction

With only 1.1 °C of warming, climate change is already causing catastrophic damage in every part of the world [1]. According to the recent estimate of the Intergovernmental Panel on Climate Change (IPCC), climate change will push 32–132 million more people into extreme poverty over the next decade [2]. Food security and food quality will be jeopardized as a result of global warming. Heatwaves and random increases in heat episodes have a wide range of negative effects on human society and the environment, including global crop production, which can be significantly affected [3], with the potential for substantial impacts on future crop demand [4]. Since episodes of heatwaves (fluctuations in daily temperature) are becoming more frequent, this is posing a direct threat to crop yield. According to a recent report by the National Aeronautics and Space Administration (NASA), climate change will affect corn and wheat production as early as 2030 [5], with corn yields expected to decline by 24% [5]. Similarly, global wheat production is estimated to be reduced by 6% for every degree Celsius rise in temperature [6], which is a substantial and worrying decline for the world’s third most important staple crop [7]. Local crop production is predicted to be affected more severely as local climates can often be subjected to more extreme changes. Overall growth and development, including fruit and grain production, of individual crop species is dependent on the surrounding soil and air temperature, distributed in the range of minimum, maximum, and optimum [8]. In addition to crops, temperature is also a key factor in determining the vegetation of a particular region [9]. High-temperature stress, or heat stress, is the rise in temperature beyond the threshold level for a period of time sufficient to cause irreversible damage to plant growth and development [10]. To date, the responses to long-term heat stress and its related molecular responses are poorly understood.

As per their ability to grow in different seasons, crops can be of two types: cool season and warm season. An increase in the temperature in the range of 32–35 °C can cause damage to most warm-season crops in the tropics and subtropics due to heat stress, whereas 25 °C is the upper threshold level to impose heat-stress-related damage on cool-season crops [8,10,11]. Heat stress, both chronic and abrupt (heat waves), affects plant growth and development. It is expected that high temperatures, such as heat waves, will affect plant growth more negatively than increases in average temperature [12], causing damage to cells or growth-stage-specific damage. For example, when the canopy of corn plants in the vegetative and reproductive stages was exposed to heatwaves for three days, the heatwave was more destructive to corn plants in the reproductive stage, decreasing their mass (16%) [13]. Similarly, an increase in temperature from ambient to 37/28 °C for 20 days resulted in decreases in the number of spikelets, early maturation, and final yield in wheat [14,15].

For optimum growth and development, plants require 17 mineral elements, including macro- and microelements [16]. Plants require elements such as nitrogen (N), phosphorus (P), potassium (K), calcium (Ca), magnesium (Mg), and sulfur (S), which are required in larger quantities and hence classified as macronutrients, whereas boron (B), chlorine (Cl), copper (Cu), iron (Fe), manganese (Mn), zinc (Zn), nickel (Ni), and molybdenum (Mo) are required in smaller amounts (micronutrients). Both macro- and micronutrients play key roles in various physiological and stress responses. Nutrient deficiency (concentrations lower than optimal levels) or toxicity (excess of any of the mineral elements) can result in physiological and metabolic disorders in plants and adversely affect plant growth [17]. Because local soil conditions fluctuate rapidly, soil nutrient availability is highly variable and often limited. Plants can uptake these nutrients successfully only as a soluble form such as ions available in the soil solution mix. Furthermore, nutrient availability in soils is affected by various factors such as soil pH, water content, redox potential, organic matter, and microorganisms [16]. Nutrient uptake in plants is also controlled by several nutrient-uptake genes specific to individual nutrients, often acting as sensors to nutrient deficiency. This aspect is discussed in detail in this review. Herein, we summarize the effects of heat stress on tissue nutrient concentration and nutrient-uptake proteins in different crop species.

**Figure 1:**
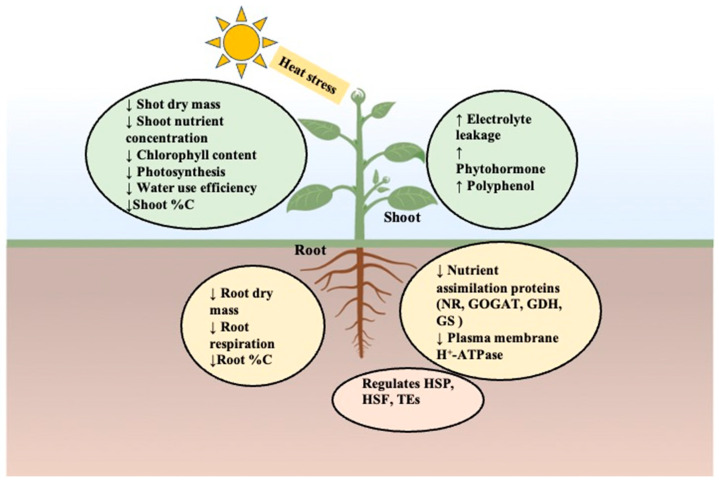
Effects of heat stress on plants. Heat stress causes decrease in growth of entire plants by affecting both root and shoot growth. Decrease in root nutrient uptake is due to the decrease in nutrient uptake proteins. Changes in root mass and nutrient uptake are also responsible for the decrease in shoot nutrient content. HSP—heat shock proteins, HSF—heat shock factors, TEs—transposon elements, NR—nitrate reductase, GOGAT—glutamine oxoglutarate aminotransferase, GDH—glutamate dehydrogenase, GS—glutamine synthetase, ↓—decrease, ↑—increase, green circle—changes in shoot system, yellow circle—changes in root system, orange circle—regulation due to heat stress.

Heat stress decreases overall plant performance and crop quality by negatively affecting growth and several physiological processes such as photosynthesis, respiration, nutrient uptake, and water relations, and it can cause membrane damage [18]. The response of plants to such abiotic stress is often complex, and plants have adapted dynamic responses at the morphological, physiological, and biochemical levels (Figure 1). While several past studies on the response of plants to heat stress emphasized a decrease in photosynthesis and biomass, more recent studies focused on phenology, grain yield, nutritional quality, phytochemicals, and essential metabolites [19,20,21,22,23,24,25,26]. Some of the common adverse effects of heat stress on plants include membrane damage, a decrease in biomass, protein denaturation, a decrease in protein concentration, and the inactivation of enzymes specific to photosynthesis and respiration [27]. The vulnerability of plants to pathogens is one of the commonly known indirect effects of heat stress on plants [28]. Several review articles are available on the effects of heat stress alone or in combination with drought or high CO_2_ [27,29,30,31,32], describing the overall effects of heat stress or interactive effects. In the current review, we primarily focus on the effects of heat stress on nutrient content, nutrient uptake, nutrient-uptake proteins, and phytochemicals, which are major factors affecting the crop quality. We briefly explain general effects before moving on to the above-mentioned specific sections.

Heat stress, alone or in combination with other abiotic factors, has profound effects on plants. Plants develop a wide range of strategies to avoid an adverse response, including a cascade of pathways, signaling, cross-talk [31], and specialized organelles [33]. In recent years, due to the advancement in proteomic and transcriptomic techniques, the roles of peroxisomes have emerged more distinctly in abiotic stress tolerance in plants [34,35]. Plant peroxisomes are also key players in cellular reactive oxygen species (ROS) homeostasis. A sharp rise in the concentration of ROS is one of the most common post-stress response mechanisms in plants to any environmental stress. If left unchecked, the accumulation of ROS could lead to oxidative damage to cells, ultimately leading to cell death [34]. The production of ROS and enhanced malondialdehyde concentration due to heat stress can cause a decrease in nutrient availability and uptake [36]. Recent studies also reported the identification of peroxisomal proteins and their crucial role in plant nutrient uptake during various abiotic stresses [37]. Thus, this review will address a new approach to investigate the response of plants to heat stress by exploring the possible role of plant peroxisomes in the context of heat stress and nutrient mobilization.

## 2. Heat Stress Decreases Plant Growth by Affecting Root Mass and Photosynthesis

The response of aerial and underground plant parts to heat stress is often complex and depends on how plants are exposed to heat stress, a factor that has differed in various studies. Generally, root growth is more sensitive than shoot growth to elevated temperatures because of the lower optimal growth temperature [20,38]. A decline in root mass might cause a decrease in shoot mass. For example, in the aeroponic culture of salad rocket plants (*Eruca sativa*), a 5 °C increase in root-zone temperature from 20 to 25 °C reduced shoot and root fresh weight with reduced net photosynthesis [21]. Several past studies, including those on non-crop species where both roots and shoots were exposed to differential air and soil temperatures, demonstrated that increased soil temperature was more detrimental than high air temperature for root and shoot growth and that shoot growth inhibition could be induced by exposing only roots to high temperatures [39,40]. The same trend was observed in tomato plants exposed to acute heat stress for six days [38]. Both root and shoot biomass were decreased by severe heat stress (42 °C), which was further accompanied by a decline in the root:shoot ratio. Higher temperature would limit root development and alter root system architecture, thus reducing the root:shoot ratio [41]. Surprisingly, several reports have confirmed that root architecture along with the root mass is impacted negatively due to heat stress, ultimately affecting the balance between numbers of lateral roots and root biomass. As a result, such changes in root architecture also negatively affect water and nutrient absorption [20,42].

Photosynthesis is another sensitive metabolic process under high-temperature stress. Heat-stress-related injuries are related to damage to photosynthetic machinery such as the denaturation of chloroplast proteins and the electron transport chain, which ultimately causes a decrease in photosynthesis [43]. However, the response also varies depending on the severity of stress. In tomato plants exposed to moderate heat stress (35 °C) for six days, surprisingly, net photosynthesis increased; nonetheless, it decreased under severe heat stress (42 °C), with decreased stomatal conductance [38]. Photochemical reactions in the thylakoid lamellae and carbon metabolism in the stroma of chloroplasts are most affected by moderate or severe heat stress, which damages the photosynthetic enzymes and proteins [44]. Heat stress could directly damage the photosynthetic apparatus, photosystems, pigments, photosynthesis-related enzyme activities, and gas exchange, leading to the inhibition of various redox and metabolic reactions and the overall process of photosynthesis [45]. A vast number of studies available on the effects of heat stress on photosynthesis either for a short period or longer duration report changes in leaf structure or anatomical changes, in addition to changes in the functioning of the photosynthetic apparatus. For example, Shen et al., 2017 [46] compared the leaf anatomy of different cultivars of Glenn Dale Azalea (*Rhododendron hybridum*) grown at 38/30 °C (heat stress) and 25/17 °C (control) for six days to study the effects of heat stress on photosynthesis. Heat stress significantly increased stomatal length (54.3%) in *Rhododendron* cultivar Liu Qiu Hong, compared with only 10.8% for cultivar ‘Lan Yin’ [46]. This size reduction and decrease in the percentage of open stomata slowed transpirational water losses, confirming the negative effects of heat stress on photosynthesis. In contrast to severe heat stress, moderate heat stress is slightly or non-damaging to PSII, even though moderate heat stress can reduce the photosynthetic rate to near zero [47]. The uptake and assimilation of some major nutrients in plants are associated with photosynthesis. For example, photosynthesis stimulates N uptake and nitrogen assimilation and is correlated with carbon status [48], primarily sugar produced by photosynthesis. Thus, the effects of heat stress are more damaging to photosynthesis by affecting biomass and, directly or indirectly, the nutrient homeostasis.

### 2.1. Effects of Heat Stress on Nutrient Content in Plants: Impact and Variability

Plant growth and development are intricately linked to the availability of mineral nutrients and the development of a well-functioning root system. Several factors, such as rhizospheric traits, root morphology, architecture, and kinetics, play key roles in regulating nutrient acquisition by plants [49]. The current review primarily focuses on the effects of heat stress on different crop species, as summarized in Table 1. During the literature survey for this current review, we found 84 relevant studies, but only 20 studies reported the effects of heat stress on nutrient concentration in different plant species (Table 1). Nutrient content in plants is directly associated with the nutritional values of fruit, vegetable, or cereal crops. We found six studies on plants that do not fall under the crop category and so we did not include them in the table. For example, when cool-season and warm-season turf grass species were subjected to heat stress (34/30 °C) for up to 28 days, concentrations of N, P, and K in cool-season turfgrass declined significantly compared to warm-season species [50]. 

The effects of heat stress on nutrient concentration vary with the exposure of plant organs to heat stress, such as the whole plant (root + shoot) or the roots alone. Similarly, the response is specific to the growth stages. For example, exposure of *Chenopodium* during anthesis to heat stress at 30 and 35 °C for 11 days led to decreased concentrations of As, Cd, Rb, and Sr in the main panicles [26]. Surprisingly, heat stress treatment of *Chenopodium* roots increased element concentration by 30%, whereas exposure of the whole plant to heat stress caused a 12% decrease in seed nutrient concentration, affecting the yield [26]. Interestingly, in tomato plants, chronic and acute heat stress only caused a decrease in total carbon (C), nitrogen (N), and iron (Fe) concentrations in roots during acute heat stress (42 °C) and a decrease in %N in the shoot system [38]. Notably, the uptake rate of boron (B) increased at moderate heat stress (35 °C) relative to other treatments, indicating that B uptake was not negatively affected by heat stress [38]. The cited work is the only study on recovery from heat stress to report no complete restoration of damaged plant-nutrient relations after six days of treatment [38].

The nutrient concentration during heat stress exhibits variation among species, elements, heat treatment types (root only or root + shoot), and duration (Table 1). For example, in *Lens culinaris* Medik, exposure to 37 °C led to decreased concentrations of zinc (Zn) and iron (Fe) compared to ambient temperature conditions [51]. Heat stress also induces localized effects on nutrient concentration in cereal crops during the grain-filling stage. For example, in bread and durum wheat genotypes (*Triticum aestivum* L. and *Triticum turgidum* subsp. *durum*) exposed to heat stress (31/20 °C, day/night), it was found that the concentrations of Fe in the culm and leaves decreased in bread wheat but increased in durum wheat. However, the opposite trend was reported as Fe concentration increased in the spike [52] (Table 1). Surprisingly, in the same study, during grain filling, the concentrations of Mn increased significantly in the shoots.

**Table 1:** Effects of heat stress on nutrient contents in different crop species.

Plant Type	Temperature (°C) Root + Shoot (R + S) or Root Only (R)	Nutrients Changed Due to Heat Stress (Increased (↑), Decreased (↓), or No Effect (∆))	References
*Chenopodium quinoa*	22, 30, and 35 (11 d, R + S)	Main panicle element content increased (↑) As, Ca, Cd, Co, Cu, Fe, K, Mn, Mo, P, Rb, S, Se, Sr, and Zn ↓ As, Cd, Rb, and Sr (main panicles), Al, B, Ni, and Rb (secondary panicles), and As, B, Cu, and Sr (tertiary panicles) ∆ Fe, K, Na, and Sr	[26]
*Eruca sativa* (cv. Arugula) and *Lactuca sativa* (cv. Canasta)	25, 38, and 42 (36 d, R + S)	↑Ca, Mg, ↓K ∆ Fe	[24]
*Lens culinaris* Medik.	27/18, 32/18 (day/night) (one crop season, R + S)	↓Zn and Fe	[51]
Indica rice varieties	19, 24 and 29 (seven days, R)	↑N during night	[22]
*Solanum lycopersicum*	25/20, 35/30, 42/37 (day/night) (six days, R + S)	↓Root %C, %N, Fe ↓shoot %N only at 42 °C	[38]
Salad Rocket (*Eruca sativa*)	25, 38 (three weeks, R)	↑Fe ↓Ca, K, Mg	[21]
*Triticum aestivum* L. and *Triticum turgidum* subsp. *durum*	25/14, 31/20 (day/night) (grain filling, R + S)	↑% and total Cu, Zn (root, shoot, spike; 2 cultivars) ↓% and total Cu, Zn (root, shoot, spike; 2 cultivars)	[53]
*Triticum aestivum* L. and *Triticum turgidum* subsp. *durum*	25/14, 31/20 (day/night) (grain filling, R + S)	↑% Fe, Mn (root, shoot, spike; 2 cultivars) ↓% and total Fe, Mn (root, shoot, spike; 2 cultivars)	[54]
*Triticum aestivum* L. and *Triticum turgidum* subsp. *durum*	25/14, 31/20 (day/night) (grain filling, R + S)	↑% and total Ca, Mg (root, shoot, spike; 2 cultivars) ↓% and total Ca, Mg (root, shoot, spike; 2 cultivars)	[52]
*Lycopersicon esculentum*, *Citrullus lanatus*	10, 25, 35 (30 d, R + S)	↑% Fe (watermelon, roots and leaves) and ↓% Fe (tomato, roots and leaves)	[55]
*Lactuca sativa*	23–38 vs. 20 (11 d, R)	↓NO_3_^−^, Ca, Cu, Fe, K, Mg, Mn, Zn	[56]
*Solanum tuberosum*	16, 20, 23, 27, 30 (120 d, R)	↓% Cu and Zn at 30 (∆)% Cu (in tuber)	[19]
*Solanum lycopersicum*	24, 27, 30, 33, 36 (9–18 d, R)	↓Mn, P (mg/plant) ∆ Zn	[57]
*Cucumis melo*	24, 27, 30, 33, 36 (9–18 d, R)	↑Mn, P, Zn (mg/plant)	[57]
*Gleditsia triacanthos*	24, 27, 30, 33, 36 (9–18 d, R)	↓Mn (mg/plant) ∆ P, Zn	[57]
*Cucumis sativus* L. cultivar ‘Sharp I’	25, 32, 35, 38 (8 and 16 d, R)	↑% B and ↓% N, P, K, Ca, Mg, Fe, Mn	[58]
*Hordeum vulgare*, *Sorghum bicolor*	25, 35 (4 h, R)	↓K, NO_3_ ion flux to the xylem	[59]
*Solanum lycopersicum*	10, 15.6, 21.1, 26.7, 32.2, 37.8 (two weeks, R)	↓N, P, K, Mg, Mn, Zn (mg/plant) ∆ B, Fe, Mo	[60]

Based on the available literature, we conclude that heat stress can have complex and adverse effects on plant-nutrient relations. Factors such as reduced root growth and biomass, as well as limitations in water and nutrient uptake, contribute to the decline in nutrient content during heat stress. Further, the decrease in nutrient acquisition during heat stress could be attributed to various factors, such as a reduction in root mass or surface area and a decrease in nutrient uptake per unit root [49]. Moreover, the choice of experimental approaches, such as whole plant versus root-only studies, and intact or detached root-based investigations, may significantly influence the overall effects of heat stress on plant-nutrient dynamics. Although research in this area is still limited, understanding the complex effects of heat stress on plant-nutrient dynamics is critical for minimizing the effects of climate change on agricultural yield and crop quality.

### 2.2. A Decrease in Nutrient Uptake during Heat Stress Is Related to Potential Damage to Nutrient-Uptake Proteins

Nutrient demand and nutrient use efficiency are two major players in root nutrient uptake. Nutrient use efficiency refers to the ability of crops to take up and utilize nutrients for optimal yields. It involves three major processes in plants: uptake, assimilation, and utilization of nutrients. However, relatively little is known about the influence of temperature on plant–nutrient interactions in terms of nutrient uptake in a changing environment [27,38]. Plant roots are often more sensitive to heat stress than shoots, which negatively impacts plant growth and productivity by reducing root growth and function, including nutrient uptake [20,27]. To maintain nutrient homeostasis, plants must regulate nutrient uptake and respond to changes in the soil as well as within the plant [61].

The uptake of most of the mineral elements is mediated by the activity of nutrient-uptake proteins in plants. Activities of nutrient-uptake proteins depend on the concentration of uptake proteins per unit root, as well as the rate of protein function [38,62]. For example, plants uptake nitrogen either as nitrate (NO_3_^−^) or ammonium (NH_4_^+^) via three different transporter proteins. Nitrate (NO_3_^−^) uptake by the root is controlled by two kinetically distinct nitrate uptake systems, the low-affinity transport system (LATS) and the high-affinity transport system (HATS). The low-affinity transport system (LATS) is encoded by the nitrate transporter 1 (NRT1) family, and HAT is encoded by the nitrate transporter 2 (*NRT2*) family [63,64]. Similarly, plants take up ammonium by using ammonium transporter genes (AMTs) only [65,66]. Phosphorus uptake in plants has been widely investigated. To date, five Pi transporter families have been isolated and characterized in plants, PHT1–5, which are involved in Pi uptake in the plant during limited phosphorus supply [67,68]. Nutrient uptake proteins have been studied in plants for micronutrients. Two major iron uptake proteins, IRT1 and FRO1-7, are the primary Fe-uptake protein in plants [69]. Proteins responsible for micronutrient uptake in plants have been studied extensively. For example, BOR1 and NIP5;1 have been reported as the primary boron (B)-uptake proteins during B deficiency [70,71]. Several studies have reported that BOR1 and NIP5;1 protein levels changed due to the combined effects of abiotic stress (increased irradiance or high CO_2_) and B deficiency [62,72]. Foliar application of zinc (Zn) increased the expression of Zn-uptake proteins (ZIPs) in plants [73]. In general, the function of these nutrient-uptake proteins is also dependent on location. For example, the nitrate uptake protein in *Arabidopsis thaliana* (AtNRT1-1) is located on the plasma membrane and the gene is expressed in the epidermis, cortex, and endodermis in mature parts of the root and helps in nitrate uptake under low nitrogen availability [74], whereas At NRT1-5 is located on the plasma membrane of the root pericycle close to the xylem and is involved in long-distance transport of nitrate from the root to the shoot [75]. The uptake of both macro- and micronutrients by the root is tightly regulated in response to changes in soil nutrient availability and demand. For example, according to the results from various studies, HATS for the various N sources are particularly responsive and display much higher flexibility than the corresponding LATS [64]. Sensing N in soil and downregulating it via feedback repression mechanisms upon reaching the optimal internal concentration are two major mechanisms that work synergistically to modulate root N uptake (external N supply or internal N demand). A similar mechanism has been reported for the uptake of boron (a micronutrient). Under B-deficient conditions, boric acid channels and borate exporters (NIP5;1 and BOR1) facilitate boron uptake and translocation to the entire plant. After reaching an adequate level, it induces downregulation of NIP5;1 and BOR1 via mRNA degradation and proteolysis via endocytosis, respectively [76].

Surprisingly, most studies on nutrient uptake in plants were conducted only by changing the nutrient concentrations either to deficiency or toxicity levels. Only a few studies are available on the status of nutrient uptake proteins during abiotic stress [38,62,72,77,78,79,80]. In all these studies, relative levels of the major nutrient-uptake proteins (NRT1, NRT2, AMT1, PHT1, BOR1, NIP5;1, and FRO1) per unit total root protein were quantified using ELISA (enzyme-linked immunosorbent assay) or Western blots [62,72] using protein-specific antibodies. Polyclonal antibodies were designed that were specific to conserved domains of certain nutrient-uptake proteins across species [62,72,77]. Of the above-mentioned seven studies, only one study reported a change in nutrient-uptake protein due to heat stress alone [38]. The rest of the studies reported combined effects of heat stress and high CO_2_ concentration [79,80]. Changes in nutrient concentration in plants upon exposure to high temperatures could be due to the effects on proteins involved in the uptake of specific nutrients. However, the molecular studies of most of the nutrient-uptake proteins suggest higher protein expression under deficiency conditions of specific nutrients compared to the optimum concentration [63,69,70,74]. When *Solanum lycopersicum* (tomato) plants were exposed to moderate heat stress (35/30 °C day/night) or severe heat (42/37 °C day/night), there was a decrease in total root protein concentration in roots in addition to a decrease in %C and %N. Heat stress both at 35 °C and 42 °C initially decreased the relative concentration (per g dry root) of all the nutrient-uptake proteins (NRT1, NRT2, AMT1, PHT1, FRO1, BOR1, and NIP5;1) compared to control plants of the same age [38]. In contrast, after six days, the relative concentrations of nutrient-uptake proteins in moderate heat-stressed (35/30 °C) plants were similar to those of control plants. The study by Giri et al., 2017 [38] also reported that severe heat stress (42/37 °C) was damaging to all tested nutrient-uptake proteins (except NIP5;1 and AMT1). This is the only study where nutrient-uptake proteins were also measured after seven days of recovery. Surprisingly, levels of these nutrient-uptake proteins had recovered similarly to control plants for all except for the iron-uptake protein FRO1. Similarly, when tomato plants were grown under high CO_2_ and moderate heat (37 °C), heat stress alone decreased the concentration of nitrate transport protein (NRT1) and AMT1, the ammonium-uptake protein [79]. While direct evidence on the effects of heat stress on individual nutrient-uptake proteins is limited, several indirect pieces of evidence address the possible effects on this factor. For example, variation in temperature from mild to high can cause alternative splicing of primary transcripts of several genes, which ultimately results in their degradation or translation to alternative protein products involved in nutrient acquisition and homeostasis [81]. Among the indirect mechanisms, here, we would like to highlight the possible role of plasma membrane H^+^ATPases (PM H^+^ATPase) due to their centrality in various physiological processes in plants [82]. The PM H^+^ ATPases help in nutrient absorption and transport by generating proton gradients and electric potential differences, which energize secondary active transport and allow nutrients to enter plant cells [83]. Interestingly, the uptake of several nutrients by roots is dependent on a proton gradient (H^+^) generated by the plasma membrane H^+^-ATPase. The plasma membrane of a cell is one of the primary sites to respond to high temperature by changing the expression of PM H^+^ATPase. For example, PM H^+^ATPase transcript level and protein abundance increased when pea plants were exposed to two different higher temperatures, 38 and 48 °C [84]. Surprisingly, when tomato plants were exposed to moderate (35/30 °C) and severe heat stress (42/37 °C), the level of PM H^+^ ATPase decreased under both heat stress conditions [38]. However, there was a close-to-normal recovery of the root H^+^-ATPase level reported in the same study when plants recovered from heat stress [38]. Recent studies have reported that increased ATPase activity promotes heat resistance in plants by improving energy status and enhancing nutrient uptake [85,86]. The PM H^+^ ATPase also modulate nutrient uptake by stimulating the nutrient-uptake protein located in the plasma membrane of root cells. Ammonium uptake, for example, is controlled by NH_4_^+^/H^+^ symporter activity because the ammonium transporter AMT1, located in the plasma membrane of root cells, and PM H^+^ ATPase promote apoplast acidification, which stimulates AMT1-mediated NH_4_^+^ transport [87]. Recent studies have reported that increased expression and activity of PM H^+^ ATPase is positively correlated with activation of PHT1 under low P availability [88], further supporting the possible indirect effects of heat stress on nutrient concentration and uptake in plants.

From these limited numbers of studies on the effects of heat stress on nutrient uptake and nutrient-uptake proteins, it can be summarized that the decrease in the level of nutrient uptake could be due to the direct heat damage to roots [20], which might decrease the production or function of nutrient-uptake proteins. Even a shorter duration of heat stress can decrease the total protein concentration and levels of nutrient-uptake and assimilatory proteins in roots of different crop types. Even if the distinct underlying mechanism is unknown, it is clear that more targeted heat-stress-induced impacts on specific nutrient absorption and transport at the transcriptional, translational, or post-translational levels cannot be ruled out. Future studies should concentrate on the effect of heat stress on nutrient-uptake genes and proteins, in particular.

## 3. Changes in Phytochemicals Influence Plant Responses to Heat Stress

Research on the effects of heat stress on phytochemicals has produced mixed results, and the lack of a consistent methodology has made comparisons between studies difficult (Table 2). In a study of five clones of (*Vitis vinifera* L.) cv. Tempranillo grapes grown at 4 °C above the ambient temperature (exact temperatures were not reported), warm temperatures significantly increased anthocyanin concentrations in the grape skins 2 weeks after mid-veraison compared to the start of the experiment without affecting anthocyanin levels at maturity (*p* < 0.05) [89]. Research comparing tomato plants (*Solanum lycopersicum* L.) exposed to 45 and 50 °C (1 h per day for 7 days) to those grown at 25 °C found a strong, positive correlation between heat stress and total phenolic content (*p* = 0.016, *r* = 0.94) [90]. Flavonoid content decreased significantly (*p* < 0.05) at 45 vs. 25 °C (≈61 vs. 31 mg QE/100 g FW); however, the fruit was not assessed in this project (only the epigeal part of the plants was studied). Scarano et al., 2020 [91] measured beta-carotene concentration in heat-stressed tomato fruit grown outdoors with temperatures greater than 32 °C for 40 days and over 35 °C for 16 days. Beta-carotene concentration increased in heat-stressed plants versus controls (*p* < 0.05). In tomato fruit grown at a mean temperature of approximately 36 ± 2 °C compared to those grown at 32.1 ± 1 °C, lycopene content decreased with increasing temperature (*p*-value not reported) [92]. Another study compared the total phenolic and flavonoid contents of tomato fruit grown in greenhouses with and without shade over four months (mean maximum monthly temperature range of 32.9 ± 3.1 to 39.1 ± 4.7 °C) to those of fruit grown in an open field (mean maximum temperature of 22.3 ± 3.9 °C) [93]. The authors reported that total phenolic content was significantly greater (*p* ≤ 0.05) for plants grown in the open field (54.3 ± 4.1 mg GAE/100 g FW) and greenhouse (52.4 ± 6.1 mg GAE/100 g FW) versus those in the shaded greenhouse (37.0 ± 4.7 mg GAE/100 g FW), as was total flavonoid content for plants grown in an open field (16 ± 5.6 mg QE/100 g FW) versus those in a shaded greenhouse (8.7 ± 1.7 mg QE/100 g FW, *p* ≤ 0.05). The authors attributed these differences to the higher ultraviolet radiation to which plants grown without shade were exposed and concluded that this may have caused greater synthesis of phenols and flavonoids [93]. Thus, in tomatoes, heat stress may affect concentrations of potentially beneficial nutrients, particularly in the fruit. Additional research with a consistent methodology will help clarify these findings.

Results have also varied among studies considering the effects of heat stress on phytochemicals found in vegetables. A study of sweet basil (*Ocimum basilicum* L.) reported that total carotenoids decreased significantly (*p* = 0.05) over 21 days in plants kept at 55 °C compared to the control at 25 °C (about 0.45 vs. 0.1 mg g^−1^ DW) [94]. Total flavonoids significantly increased (8.72 ± 0.59 at 25 °C vs. 13.69 ± 0.54 mg eq “C” g^−1^ DW at 55 °C). In an experiment studying salad greens, arugula (*Eruca sativa*) and Canasta (*Lactuca sativa*), plant samples were randomized to three groups and grown at various ambient temperatures [24]. In each experimental group, the roots were exposed to one of three temperature conditions: 25 °C for 36 d, 25 and 42 °C for 20 and 16 d, respectively; and 25, 38, and 42 °C for 10, 10, and 16 days, respectively. Total phenolics decreased for all plants as storage time increased (*p* < 0.06) [24]. Despite this decline, compared to the control plants, those exposed to heat stress showed greater phenolic compounds (*p* < 0.05). Finally, in cauliflower (*Brassica oleracea* var. *botrytis*), research has examined the effects of a nutrient solution in promoting adaptations to heat stress [95]. During the heat stress period, the temperature varied from 43 °C by day to 30 °C at night. Treatment with a 50:50 nutrient solution of NO_3_^−^ and NH_4_^+^ and foliar application of 2.5 mM putrescine prior to 72 h of the heat stress condition increased total phenolic content and antioxidant activity (total phenolic content of 574.8 μg GAE g^−1^ FW vs. 80.5 μg GAE g^−1^ FW in the control treated with NO_3_^−^ only and total antioxidant activity of 49.00 vs. 339.26 μmol Trolox g^−1^ DW in the control, *p* ≤ 0.05 for all) [95]. The application of nutrient solutions and their ability to better enable plants to withstand heat stress deserves further consideration, given their potential to enhance nutrient retention in food crops.

Although grains are an important source of carbohydrates, fiber, and other nutrients, a limited number of experiments have assessed the effects of heat on the antioxidants they contain. A study of Durum wheat (*Triticum turgidum* L.) grown at 37 °C during the day and 17 °C at night for 5 days reported significantly increased anthocyanin levels in the T1303 Ethiopian purple genotype, which has a high anthocyanin content in its grains, compared to a high-carotenoid yellow genotype (“Primadur”) (*p* < 0.05). Heat stress did not affect carotenoids in either genotype. Research with quinoa (*Chenopodium quinoa* Wild) found that the effects of heat on antioxidant capacity may also vary by cultivar [97], suggesting that differences between cultivars (e.g., color and response to heat) will be another important factor to consider in the future when selecting seeds for breeding.

Nutrient deficiency often stimulates the production of phenolics in plant tissues [98]. Several studies have reported that exposure of plants to higher temperature increases a phenolic-rich root exudate that solubilizes the different nutrients from unavailable sources to facilitate their uptake by plants [99]. Thus, although heat stress may increase phytochemical concentrations in some plants, the mixed results from other studies suggest that additional research in this area is needed to correlate with changes in plant-nutrient relations during heat stress.

## 4. Cell Organelle and Heat Stress: Potential Role of Peroxisomes in Heat Stress Amelioration

In eukaryotic cells, various membrane-bounded compartments operate and coordinate specific biochemical processes. They can be surrounded either by a single or double membrane or, in certain circumstances, they lack a membrane. The peroxisome is one such compartment surrounded by a single membrane. Peroxisomes are present in nearly all eukaryotes and perform diverse functions. Peroxisomes exhibit functional plasticity with developmental stages, nevertheless, their primary functions are of the oxidative type [100,101]. Unlike other major cell organelles, such as mitochondria and chloroplasts, peroxisomes do not contain any genome of their own; hence, all the peroxisomal proteins are synthesized on cytosolic ribosomes and imported in a signal-dependent manner. However, the modus operandi for the import of peroxisomal membrane and matrix proteins is entirely different. Peroxisome membrane proteins are imported either by direct delivery to the peroxisome membrane or mediated by endoplasmic reticulum [102], while matrix proteins are primarily imported by peroxisomal targeting signal (PTS) type 1 or PTS2. The PTS1 and PTS2 proteins are recognized by their respective cytosolic receptors peroxin (PEX) 5 and PEX7, followed by import to the peroxisome with the help of other PEX family proteins. The PTS1 is present at the C-terminus of the protein while the PTS2 is present at the N-terminus of proteins [103,104].

The generation of reactive oxygen species (ROS) due to stress conditions is a common phenomenon in plants and it is very important to regulate ROS homeostasis. If ROS production is left unchecked, it may be detrimental for the cell. Peroxisomes in association with mitochondria and chloroplasts form the trinodal center for cellular ROS homeostasis [105,106,107,108]. Peroxisomes have also been demonstrated to contain regulatory proteins such as kinases, phosphatases, and heat shock proteins, which play a crucial role in abiotic stress signal transduction [109,110]. The recent advances in bioinformatics, genomics, and proteomics research have demonstrated that the peroxisomal functions are linked with ROS and reactive nitrogen species (RNS) homeostasis, as well as a key apparatus for redox signaling during abiotic stress conditions [110,111,112,113,114,115].

**Figure 2:**
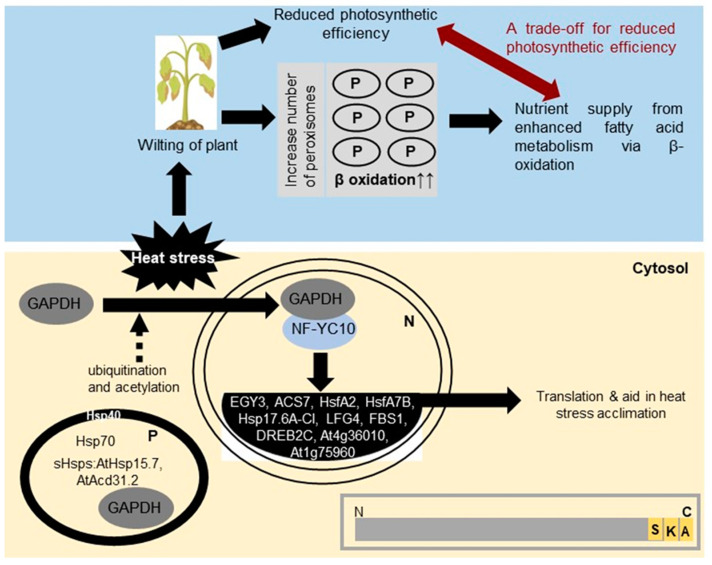
Overview of peroxisomal involvement in heat stress acclimation: Heat stress enhances the number of peroxisomes, leading to an increased rate of beta-oxidation, making more fatty acid breakdown products available for metabolism. The heat stress also leads to the movement of GAPDH protein to be translocated to the nucleus, where it binds with NF-YC10 transcription factor, leading to the upregulation of a battery of genes, which ultimately aids in heat stress acclimation. GAPDH has been proposed to localize in the cytosol, peroxisome, and nucleus. The light yellow area shows the cellular-level changes while the blue area shows the organismal-level changes. The grey-colored box in the bottom right-hand corner shows a diagrammatic representation of GAPDH protein (grey-colored rectangle), demonstrating its putative PTS1 domain (marked as three yellow strips for the three C-terminus amino acids representing the putative PTS1). The broken arrow denotes the putative involvement of ubiquitination and acetylation in the translocation of GAPDH from the cytosol to the nucleus. N—nucleus, P—peroxisome, GAPDH—glyceraldehyde-3-phosphate dehydrogenase, NF-YC10—nuclear factor Y subunit, EGY3—ethylene-dependent gravitropism-deficient and yellow-green-LIKE3, ACS7—1-amino-cyclopropane-1-carboxylate synthase7, Hsf—heat shock transcription factor, LFG4—LIFEGUARD4, a Bax inhibitor 1 family protein, FBS1—F-Box stress induced 1, DREB2C—dehydration-responsive element binding protein 2C, At4g36010 and At1g75960—accession number from TAIR (the Arabidopsis information resource), for which no annotation has yet been given.

Very few studies are available on the role of peroxisomes in heat stress tolerance in plants. The involvement of glyceraldehyde-3-phosphate dehydrogenase (GAPDH) in heat stress amelioration in *Arabidopsis thaliana* (At) was reported by Kim et al., 2020 [116]. AtGAPDH is primarily a glycolytic enzyme catalyzing the conversion of glyceraldehyde-3-phosphate to 1,3-bisphosphoglycerate. However, Kim et al., 2020 [116] demonstrated a moonlighting effect of the enzyme GAPDH, where it was demonstrated that under stress conditions, GAPDH moves to the nucleus and binds with the nuclear factor Y subunit C10 (NF-YC10). The binding of GAPDH with transcription factor NF-YC10 promotes the expression of heat-stress-inducible genes and imparts heat stress tolerance to *A*. *thaliana* plants. The excess accumulation of GAPDH in the nucleus was also demonstrated. Furthermore, the overexpression and knockout of GAPDH were linked with enhanced heat stress tolerance and sensitivity, respectively, in *A. thaliana* plants. The overexpression of AtGAPDH followed by heat stress tolerance of *A*. *thaliana* was linked to the upregulation of a subset of heat-inducible genes. A 3–4-fold increase was observed under heat stress in the case of *EGY3* (ethylene-dependent gravitropism-deficient and yellow-green-LIKE3, At1G75860) and *ACS7* (1-amino-cyclopropane-1-carboxylate synthase 7). Furthermore, an eight-fold increase in the expression of heat-stress-related genes—heat shock transcription factor (*Hsf*) *A2*, *HsfA7B*, *Hsp17.6A-CI*, At4g36010 (encoding a pathogenesis-related thaumatin superfamily protein), *LFG4* (LIFEGUARD4, a Bax inhibitor 1 family protein), *FBS1* (F-Box stress induced 1), *DREB2C* (dehydration-responsive element binding protein 2C), and At1g75960 (encoding an AMP-dependent synthetase and ligase family protein)—was observed [116] (Figure 2). GAPDH is a cytosolic enzyme, and Kim et al., 2020 [116] used the cytosolic isoform. However, the same isoform was also found in peroxisomal fractions in proteomic studies [117]. It is presumed that GAPDH (At1g13440) is being targeted to peroxisomes via a PTS1, represented by SKA> (“>” denotes the end of the polypeptide chain), which is a non-canonical type of PTS1, suggesting that the protein GAPDH may be targeted to three subcellular sites—the cytosol, nucleus, and peroxisome (Figure 2). It has been observed that lysine ubiquitination by E3 ubiquitin-ligase and lysine acetylation could facilitate the nuclear transport of the protein, and GAPDH has been demonstrated to undergo both the post-translational modifications [118,119]. GAPDH has also been demonstrated to undergo S-nitrosylation, S-sulfhydration, and S-glutathionylation, suggesting that GAPDH is susceptible to multiple post-translational modifications [120,121], which could facilitate different subcellular targeting.

As explained above, the expression of heat shock proteins (Hsps) is upregulated under high temperature [122]. They facilitate the refolding of partially denatured proteins or the folding of newly made proteins. Under stress conditions, protein denaturation increases and hence the activity of Hsps also increases. Wimmer et al., 1997 [123] and Diefenbach and Kindl 2000 [124] demonstrated the localization of Hsp70 and DnaJ (Hsp40) homolog from *Citrullus vulgaris* and *Cucumis sativus*, respectively, to peroxisomes. Hsp70 is targeted to the peroxisomal matrix via a PTS2 represented by RTx_5_KL [123], while DnaJ is a peroxisomal membrane protein [124]. Another class of Hsps, the small Hsps (sHsps), is known to prevent the aggregation of misfolded proteins, formed as a result of stress conditions [125,126]. The sHsps range from 16 to 42 kDa and contain an α-crystalline domain of about 90 amino acid residues present at the C-terminus [127]. Ma et al., 2006 [128] reported the peroxisomal localization of two sHsps, AtHsp15.7 (At5g37670) and AtAcd (alpha crystalline domain, At1g06460) 31.2. AtHsp15.7 is targeted to the peroxisome via a PTS1 represented by SKL>, while AtAcd31.2 uses a PTS2 represented by RLx_5_HF. *AtAcd31.2* was found to be constitutively expressed, while *AtHsp15.7* was primarily induced by heat and oxidative stress [128]. The overexpression of sHsps could be helpful in imparting abiotic stress tolerance to plants.

Differential behavior of the same candidate protein under short-term (42 °C for 30–60 min) and long-term heat stress (37 °C for several days) was also observed. In *A*. *thaliana*, long-term heat stress led to hyper-expression of the catalase (*CAT*)*2* gene; however, no effect on the expression of *CAT2* was observed upon short-term heat stress [129]. The *cat2* mutants were found to be hypersensitive to long-term heat stress while tolerant to short-term heat stress. Furthermore, it was also observed that hydrogen peroxide (H_2_O_2_) increased in a time-dependent manner in response to long-term heat stress while it remained nearly unaltered under short-term heat stress [129]. The studies conducted here present a crucial account of variation in the response of plants under different forms of heat stress conditions and could be critical in designing heat-stress-tolerant plants.

Heat stress has also been demonstrated to affect the reproductive tissues, resulting in the poor fertility of plants [130,131,132]. A detailed comparative proteomic analysis of the anther (male reproductive structure) amongst heat-sensitive, moderately heat-tolerant, and heat-tolerant *O*. *sativa* varieties revealed a distinct set of proteins being up- and downregulated. The proteins functioned in hormone biosynthesis, photosynthesis, stress tolerance, signal transduction, redox potential, transporters, RNA regulation, protein synthesis, and carbohydrate, lipid, and amino acid metabolism. The list includes two peroxisomal proteins as well, namely an AMP-binding enzyme (LOC_Os09g21230.1) involved in lipid metabolism and a putative methyltransferase (LOC_Os04g59590.1) involved in stress responses. The former was found to be induced to a higher extent while the latter was moderately upregulated [130]. The peroxisomal localization of both proteins was predicted on the basis of the PTS1 prediction algorithm [103,133]. Both the proteins were predicted to be targeted to the peroxisome via a PTS1. In the cases of AMP binding protein and methyl transferase, the PTS1 was represented by a canonical SKI> and AKL>, respectively. Furthermore, heat stress was also demonstrated to negatively affect the female reproductive structure by causing irreversible anatomical and physiological changes, leading to a reduction in the number of ovules. The reduction in the number of ovules was found to be related to disruption in the normal development of the ovary. These forms of damage were found to be pronounced in the heat-stress-sensitive varieties as compared to the heat-stress-tolerant varieties [131].

The transport of metabolites and signaling molecules across the intracellular membranal network is a crucial phenomenon and is essential for maintaining cellular processes. Peroxisomes are no exception to this. The membrane of peroxisomes primarily has two types of transporters—the pore-forming diffusion channels referred to as non-selective transporters, and carrier proteins with high specificity. The former allows the passage of small hydrophilic molecules up to 300–400 Da, such as amino acids, while the latter is primarily involved in the transport of larger molecules, such as NAD+ [134,135,136]. The current status of knowledge about the peroxisomal transport proteins is rather limited, and knowledge on their involvement and behavior in stress conditions is all the scarcer. In one study we could find, Wu et al. (2005) [137] reported a peroxisomal channel protein in smooth bromegrass (*Bromus inermis*) that could be involved in fatty acid or succinate transport, and its expression was found to be modulated in response to plant hormone abscisic acid, cold, and dehydration stress.

### Nutrient Availability during Heat Stress: A Peroxisomal Perspective

An increase in the number of peroxisomes upon heat stress in the *Chenopodium quinoa* plant, which correlated positively with H_2_O_2_ content in leaves and negatively with the yield of the crop, has also been reported [138]. New peroxisomes arise either de novo from the endoplasmic reticulum or form via the fission of existing peroxisomes [35,139]. Pex11 has been considered a marker for the fission process [140,141], which functions together with the FIS1 gene family [142]. The upregulation in the expression of *Pex11A*, *Pex11B,* and *Fis1A* in the *Chenopodium quinoa* plant has been observed during heat stress conditions [138], which mediate the enhanced number of peroxisomes (Figure 2). Increased temperature has also been linked with reduced levels of PEX5, and it magnifies the peroxisomal defects in *pex4* mutant lines, which have impaired beta-oxidation [143]. It has been observed that high temperature leads to a reduction in the photosynthetic efficiency of plants [144], which ultimately leads to lower food production, meaning that cells turn toward the stored food materials. Lipids and fats constitute one of the most significant food reserves in organisms. β-oxidation is the primary pathway that converts lipids/fats to acetyl-CoA, which ultimately enters the Krebs cycle and meets the required energy demand. Hence, we believe that at higher temperatures, the increase in the number of peroxisomes could be a trade-off for the reduced level of peroxisomal functions, such as β-oxidation. This makes peroxisomes a very important organelle in relation to the nutrient supply to plants under stress conditions. Kataya et al., 2016 [145] reported the peroxisomal localization of a purple acid phosphatase (PAP) protein. PAPs have been demonstrated to play a significant role in the phosphate starvation of plants. The expression of PAP was found to be upregulated upon phosphate starvation [146]. Phosphate being a very critical nutrient for plants makes PAP a very significant enzyme under stress conditions.

## 5. Conclusions and Future Perspectives

**Figure 3:**
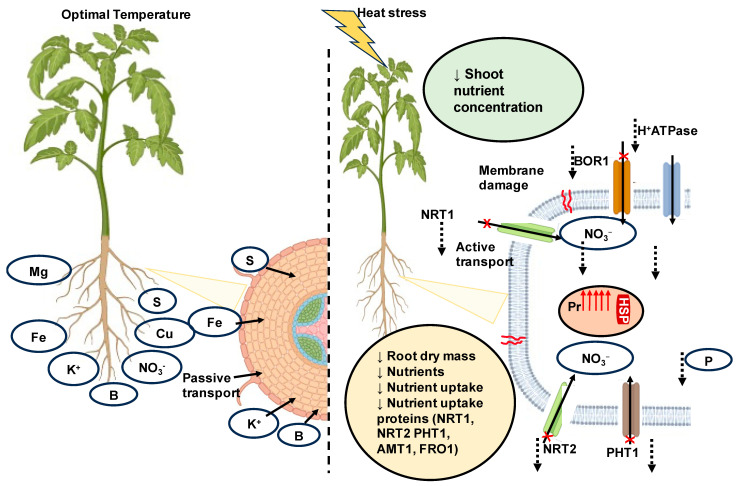
Effects of heat stress on nutrient uptake and nutrient-uptake proteins. Under optimal conditions, nutrient uptake occurs via passive transport as nutrients diffused into root hairs, whereas under limited availability, nutrients enter through active transport. Heat stress causes a decrease in root nutrient uptake due to the decrease in nutrient-uptake proteins, root growth, and damage. Changes in root mass and nutrient uptake are also responsible for the decrease in shoot nutrient content, ultimately decreasing the nutrient concentrations in the shoot system. Damage of cell membrane due to heat stress potentially damages the nutrient uptake proteins or NRT1—low-affinity (NO_3_^−^) transporter, NRT2—high-affinity (NO_3_^−^) transporter, AMT1 ammonium (NH_4_^+^) transporter protein, PHT1—phosphorus uptake protein, FRO1—iron uptake protein, Pr—peroxisome, red upward arrow indicates increase in number of peroxisomes. Heat stress leads to chlorophyll degradation, to which one of the responses of the cell is an increase in the number of peroxisomes, leading to enhanced beta oxidation, aiding in energy production. The red barrel-shaped structure indicates an accumulation of heat shock proteins (HSPs), which help in acquiring native confirmation to denatured proteins. ↓—decrease, ↑—increase, green circle—changes in shoot system, yellow circle—changes in root system, orange circle—regulated due to heat stress, X—damage, 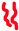 —membrane damage.

As a consequence of global warming, sudden increases in temperature due to heat waves may become more frequent and negatively impact plant function. The response of plants may vary depending on the plant species and type. In the studies we reviewed, the effects of warming exhibited the greatest impacts on plant growth and development at different growth stages, such as the vegetative, reproductive, and grain-filling stages, ultimately affecting grain quality and overall crop quality and yield. The deleterious effects of heat stress on plants resulted in the disruption of enzymes responsible for nitrogen metabolism, including those involved in the assimilation of nitrate and ammonium, affecting several key metabolic processes in plants. While numerous review articles have explored the broader impact of heat stress on plants, including physiological, biochemical, and molecular mechanisms and the regulation of HSPs, there is a notable lack of studies addressing the specific effects of heat stress on crop quality in terms of nutrient content, uptake, and the underlying mechanisms of gene regulation specific to nutrient uptake during heat stress. Despite the lack of a defined mechanism for the effects of heat stress on nutrient uptake and transport, this review concludes that heat stress adversely affects nutrient concentration in plants by affecting nutrient uptake and translocation. Decreases in nutrient acquisition with heat stress could potentially be caused by several factors, including a decrease in root mass or surface area and/or a decrease in nutrient uptake per unit root, as well as reduced photosynthetic efficiency. Furthermore, it is evident that reductions in root growth and the rate at which plants absorb nutrients are the result of heat-stress-induced cell damage in the root. This damage ultimately leads to a decline in root growth and the overall concentration of proteins, including a decrease in the levels of proteins responsible for nutrient uptake, and potentially affects the activity of specific uptake proteins, such as their transport or reaction rates (Figure 3). However, when we evaluated the effects on phytochemicals, we found mixed results, and the lack of a consistent methodology made comparisons between studies difficult. With the occurrence of heat waves due to climate change becoming more frequent, this can have an adverse effect on crop yield and quality. Future research must comprehensively investigate nutrient-uptake proteins using molecular analysis, with a specific emphasis on understanding their regulation mechanisms under heat stress conditions. This research should aim to elucidate the impact of such regulation on nutrient concentration and crop quality. Expanding our knowledge of cell signaling mechanisms, such as the role of peroxisomes, in nutrient mobilization via β-oxidation during stress conditions— is a significant avenue for future research on the effects of heat stress on crops.

## Figures and Tables

**Table 2:** Effects of heat stress on phytochemical concentrations in different crop species.

Plant Type	Temperatures (°C)	Responses Measured	Summary of Heat Effects on Phytochemicals	References
*Solanum lycopersicum* L.	25, 45, or 50 (1 h per d for 7 d)	Phenolic and flavonoid content	Positive correlation with total phenolic content. Decreased flavonoid content.	[90]
*Lycopersicum esculentum* Mill.	25	Total phenolic and flavonoid content	Greater total phenolic and flavonoid content for plants grown in an open field vs. a shaded greenhouse.	[93]
*Ocimum basilicum* L.	25, 35, 45, 55 (15 and 21 d)	Total carotenoids and flavonoids	Decreased total carotenoids. Increased total flavonoids.	[94]
*Vitis vinifera* L.	4 degrees above an unspecified ambient temperature	Anthocyanin concentrations	Increased anthocyanin concentrations 2 weeks after mid-veraison. No effect at maturity.	[89]
*B. alboglabra* *Brassica oleracea* var. *botrytis*	30/43 for 72 h	Total phenolic and antioxidant content	Treatment with nutrient solution beforehand increased total phenolic content and antioxidant activity.	[95]
*Triticum turgidum* L.	37 during the day and 17 at night for 5 days	Anthocyanin synthesis and carotenoids	Increased anthocyanins. No effect on carotenoids.	[96]
*Chenopodium quinoa* Wild	Mean maximum temperature of about 22 over 3 y	Total polyphenols and flavonoids	Antioxidant content varied by cultivar.	[97]
*Eruca sativa* *Lactuca sativa*	25 (36 d),25/42 (20/16 d), 25/38/42 (10, 10, 16 d)	Total phenolic concentration	Increased total phenolic compounds.	[24]
*Solanum lycopersicum* L.	>32 for >40 d; >35 on 16 d	Phytonutrient concentration	Increased beta-carotene concentration.	[91]
*Solanum lycopersicum* L.	32.1/24 ± 1 (mean day/night temperature) vs. 36 ± 2 (heat stress condition)	Lycopene concentration	Decreased lycopene.	[92]
